# Research Progress on Multiplexed Pathogen Detection Using Optical Biosensors

**DOI:** 10.3390/bios15060378

**Published:** 2025-06-12

**Authors:** Yue Wu, Xing Xu, Yinchu Zhu, Jiaxin Wan, Xingbo Wang, Xin Zhou, Xiangjun Li, Weidong Zhou

**Affiliations:** 1 State Key Laboratory for Quality and Safety of Agro-Products, Institute of Animal Husbandry and Veterinary Science, Zhejiang Academy of Agricultural Sciences, Hangzhou 310021, China; xvxing@zaas.ac.cn (X.X.); zhuyinchu@zaas.ac.cn (Y.Z.); wangxingbo@zaas.ac.cn (X.W.); zhouxin@zaas.ac.cn (X.Z.); 2 College of Biological and Environmental Science, Zhejiang Wanli University, Ningbo 315100, China; wjxin2023@163.com; 3 College of Information Engineering, China Jiliang University, Hangzhou 314423, China; xiangjun_li@cjlu.edu.cn

**Keywords:** multiple pathogens, optical biosensors, microfluidic devices, nucleic acid amplification, nanomaterials

## Abstract

The rapid and precise identification of multiple pathogens is critical for ensuring food safety, controlling epidemics, diagnosing diseases, and monitoring the environment. However, traditional detection methods are hindered by complex workflows, the need for skilled operators, and reliance on sophisticated equipment, making them unsuitable for rapid, on-site testing. Optical biosensors, known for their rapid analysis, portability, high sensitivity, and multiplexing capabilities, offer a promising solution for simultaneous multi-pathogenic identification. This paper explores recent advancements in the utilization of optical biosensors for multiple pathogenic detection. First, it provides an overview of key sensing principles, focusing on colorimetric, fluorescence-based, surface-enhanced Raman scattering (SERS), and surface plasmon resonance (SPR) techniques, as well as their applications in pathogenic detection. Then, the research progress and practical applications of optical biosensors for multiplex pathogenic detection are discussed in detail from three perspectives: microfluidic devices, nucleic acid amplification technology (NAAT), and nanomaterials. Finally, the challenges presented by optical biosensing technologies in multi-pathogen detection are discussed, along with future prospects and potential innovations in the field.

## 1. Introduction

The global COVID-19 pandemic has once again highlighted the persistent threat posed by infectious diseases, despite significant advancements in life sciences [1,2,3]. Contagious infections continue to be a leading cause of global mortality. Pathogens, including microorganisms such as fungi, protozoa, and bacteria, as well as molecular infectious agents such as viruses and prions, are responsible for various diseases. Each year, foodborne, waterborne, and airborne pathogens cause human infection via diverse transmission routes, resulting in over 15 million deaths globally [4,5,6,7]. Pathogens vary significantly in characteristics such as virulence, infectivity, transmission modes, and infectious doses. For instance, data by the World Health Organization (WHO) indicate an annual incidence of about 600 million foodborne infections, leading to 420,000 deaths [8]. Concurrently, the world is grappling with the COVID-19 pandemic, a global crisis with evolving data on its virulence and infectious dose. To date, COVID-19 has claimed the lives of more than 3.5 million people worldwide [9].

Many techniques have been proposed for pathogenic detection, including traditional virus isolation and culture methods, the polymerase chain reaction (PCR), and enzyme-linked immunosorbent assays (ELISA) [10]. However, the complicated procedure, long time to procure results, and requirement for well-trained professionals hinder further applications for current pathogen detection technologies. Culture-based methods require 2–3 days for definitive results. Immunoassays (e.g., ELISA) suffer from cross-reactivity (false-positive rate > 15%) and antibody affinity issues. PCR demands precise thermal cycling and skilled operators [11].

Biosensors have gained prominence in recent years for pathogenic identification. Information from various fields, including electronics, materials, physics, chemistry, and biology, are utilized to construct rapid, inexpensive biosensors with high sensitivity and selectivity. Optical biosensors address these challenges through real-time signal readout, miniaturized device design, and multiplex detection capabilities. These biosensors offer advantages such as high throughput and sensitivity, as well as rapid response times. Previous works have reviewed the use of optical biosensors for ecological monitoring, food safety, drug discovery, and clinical diagnosis [12,13,14,15]. Recent years have seen the emergence of pathogen detection methods based on optical biosensors [16,17,18,19,20], showing considerable potential due to their high sensitivity, fast detection speed, and easy integration. However, the discovery of more pathogens increases the demand for multi-pathogen detection in various fields. The technology for detecting individual pathogens is both time-consuming and expensive. Therefore, these methods are unsuitable for the early identification of mixed infections and the detection of various known and emerging contagious pathogens. Multi-pathogen detection technology can identify several pathogens simultaneously, presenting advantages such as high throughput capability and minimal loss, which increases identification efficiency [21,22,23]. For example, digital microfluidics technology can achieve droplet movement, mixing, separation, or distribution by precisely controlling discrete droplets on an electrode array. By constructing integrated biosensors for sample preparation, reaction, and detection, detection of multiple pathogens can be achieved [24].

**Figure 1:**
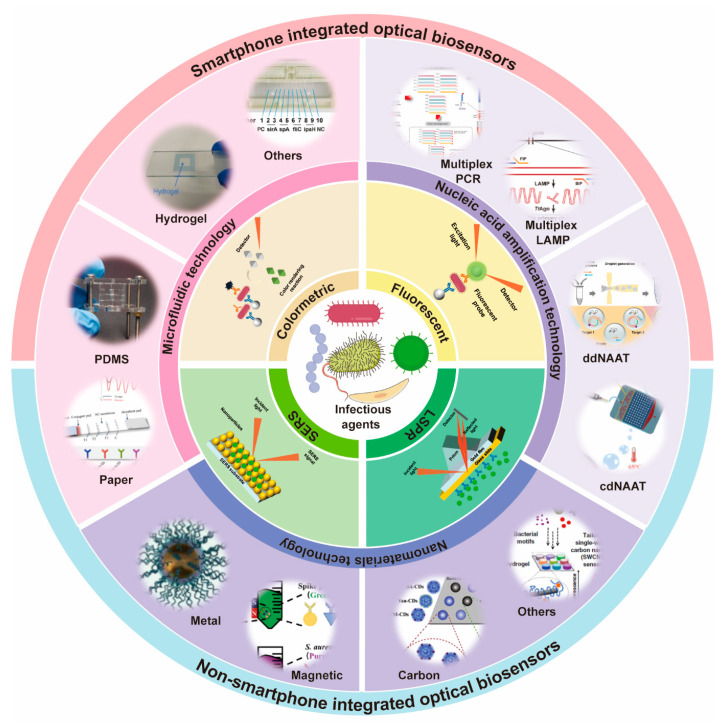
Schematic illustration of multiple pathogen detection based on optical biosensors and the recent progress focus on microfluidic, nucleic acid amplification, and nanomaterials technology. Reproduced with permission from Ref. [25]. Copyright 2025, Elsevier. Reproduced with permission from Ref. [26]. Copyright 2022, Elsevier. Reproduced with permission from Ref. [27]. Copyright 2022, Elsevier. Reproduced with permission from Ref. [28]. Copyright 2022, Elsevier. Reproduced with permission from Ref. [29]. Copyright 2022, Elsevier. Reproduced with permission from Ref. [30]. Copyright 2024, Elsevier. Reproduced with permission from Ref. [31]. Copyright 2020, Elsevier. Reproduced with permission from Ref. [32]. Copyright 2022, Elsevier.

The rapid, sensitive identification of multiple pathogens is vital for infectious disease treatment and disease transmission regulation. This article systematically reviews the research progress in recent years on optical biosensors for multiple pathogen detection (Figure 1). Section 2 introduces the main optical biosensors commonly used for pathogen detection, including their principles, advantages, and limitations. Section 3 delineates the recent research regarding the optical biosensors used for multiple pathogen detection from three perspectives: microfluidic devices, nucleic acid amplification technology (NAAT), and nanomaterials. Section 4 summarizes the current uses of optical biosensors for pathogen identification in POCT. Section 5 discusses the challenges and future prospects of using optical biosensors for multiple pathogen identification. The objective of this review is to offer insight into multiple pathogen detection and optical biosensors, aiming to enhance identification speed and accuracy. This will ultimately promote research on early infectious disease diagnosis and management to improve public health.

## 2. Overview of Optical Biosensors

Optical biosensors are analytical devices consisting of a biorecognition element and a sensing device. The working principle of optical biosensors involves changes in optical properties such as absorption, reflection, transmission, fluorescence, and scattering, which are caused by the crosstalk between the analyte and the receptor molecules on the surface of the substrate. The light output signal can be used to quantitatively analyze and record the target pathogen. The sensing ability of optical devices is achieved via labeling or label-free techniques. The label-free approach involves a simple interaction between the analyte and the transducer surface to facilitate signal detection, while label-based sensing employs specific optical labels to tag the target analyte, generating various optical phenomena to obtain the subsequent signal. These two strategies are extensively used in optical biosensing for the accurate and clear communication of information regarding the interaction between the biosensing element and the target, emphasizing the versatility of optical biosensors compared to alternative sensing platforms. Optical biosensors are typically divided into colorimetric, fluorescence, SERS, and SPR biosensors [33].

### 2.1. Colorimetric Optical Biosensors

**Figure 2:**
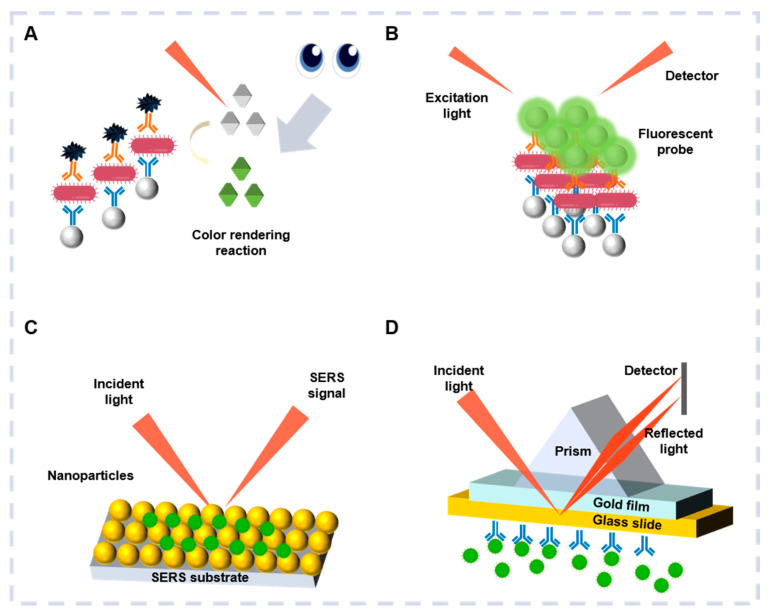
(**A**) Schematic illustration of colorimetric-based optical biosensor. (**B**) Schematic illustration of fluorescence-based optical biosensor. (**C**) Schematic illustration of SERS-based optical biosensor. (**D**) Schematic illustration of SPR-based optical biosensor.

Colorimetric sensors are capable of rapid pathogenic detection by producing color changes via physical, chemical, and biochemical reactions to identify the presence and concentrations of analytes (Figure 2A) [34]. It offers benefits, including high visibility, simplicity, rapid reading speed, and cost-efficiency [35]. Colorimetric and optical biosensors are often combined, increasing the application potential for environmental monitoring, food safety, biomedicine, and clinical diagnosis [36].

Colorimetric sensing using organic dyes can be employed for rapid on-site pathogen detection. Color changes can be readily observed with the naked eye without the need for complex instruments and equipment. Trinh et al. reported a slidable paper-embedded plastic optical biosensor utilizing colorimetric detection. The researchers designed specific primer sets for *Salmonella*, *Staphylococcus aureus*, and *Escherichia coli O157:H7* and used loop-mediated isothermal amplification (LAMP) technology to amplify these three pathogens, respectively. A specific primer mixture for different pathogens is pre-stored on each paper. When the sample is introduced, the paper in different areas is contacted with the sample in turn through a sliding device, so as to achieve simultaneous amplification and detection of the three pathogens. Finally, the color change of magenta is observed to determine whether the target pathogen exists: if an area shows dark purple, it means that the pathogen corresponding to the area is present in the sample; if there is no color change, it means that the pathogen does not exist. This method is simple and fast, and it can effectively distinguish three common foodborne pathogens [37].

Colorimetric sensors based on enzymatic reactions represent another approach to pathogenic identification. Chen et al. constructed an optical biosensor based on dual enzyme-induced colorimetry, using alkaline phosphatase for L-ascorbic acid 2-magnesium phosphate hydrate decomposition to generate ascorbic acid. The ascorbic acid reduced manganese dioxide nanoflowers to inhibit 3,3′,5,5′-tetramethylbenzidine oxidation, which prevented gold nanorod etching and resulted in significant color changes. LDA can improve the selectivity of the sensor for specific analytes by projecting the raw data into a low-dimensional space. UV–visible absorption spectral signals were combined with LDA for the simultaneous and accurate identification of five foodborne pathogens, including *Listeria monocytogenes* (*L. monocytogenes*), *Salmonella*, *E. coli*, *S. aureus*, and *Vibrio parahaemolyticus* [38].

Furthermore, multiple pathogens can also be detected simultaneously by utilizing nanoparticles to identify color changes. Wen et al. introduced an achromatic colorimetric biosensor based on magnetically separated coupled plasmon nanoparticles. Red gold nanoparticles (AuNPs) targeting SARS-CoV-2, yellow silver nanoparticles targeting *S. aureus*, and blue silver triangle nanoparticles targeting *Salmonella* were synthesized, followed by the addition of three corresponding magnetic probes, which combined with the identified target pathogens in conjunction with color reporter particles to facilitate sandwich complex formation, which was eliminated using magnetic separation. This generated color changes in the supernatant, with individual infections exhibiting distinct hues, facilitating the simultaneous identification of multiple pathogens [39]. Zhang et al. developed a colorimetric biosensor using nanoarrays. Capillary-assisted pre-concentrations were employed to enhance optical signal amplification via the nanoarrays. After capturing the target bacteria, the obvious color changes observed in the optical image allowed direct colorimetric analysis. The sensor demonstrated effective recognition and specificity for various bacteria, including *S. aureus* and *E. coli*, with an identification time below 10 min and a 10 CFU/mL limit of detection (LOD) [40].

### 2.2. Optical Biosensors Based on Fluorescence

Due to their rapid visualization and real-time monitoring capacity, fluorescent biosensors are commonly used as optical biosensors for pathogenic identification [41]. The pathogens targeted by fluorescent biosensors are labeled via the binding between a fluorescent material and the recognition element, emitting fluorescence after specific stimulation (Figure 2B) [42].

Fluorescent labels are essential components for developing fluorescent biosensors for pathogenic detection and include organic fluorescent dyes, such as carboxylic fluorescein, isothiocyanate fluorescein, and acridine orange. Contrarily, ratio-type fluorescence probes measure changes in target-induced emission intensity across two or more distinct wavelengths, providing inherent self-calibration that mitigates interference from extraneous factors unrelated to the target. This approach enhances detection sensitivity and expands the dynamic linear range. Several studies have successfully developed various ratio fluorescence probes for pathogen identification. Svechkarev et al. devised a ratiometric fluorescence sensor array, using the excited-state intramolecular proton transfer attributes of 3-hydroxyflavone derivatives to obtain a dual-channel ratiometric response. For example, the fluorescence emission spectrum of *Staphylococcus aureus* shows that N emission is strong at 485 nm and T emission is weak at 575 nm. The fluorescence emission spectrum of *Escherichia coli* shows that N emission is weak at 485 nm and T emission is strong at 575 nm. The sensor can discern eight distinct bacterial species and their Gram-staining characteristics via linear discriminant analysis [43]. Some studies have used bacterial cells with substantial, uniform molecular dimensions at the micrometer scale as effective signal amplification carriers to load various signal molecules. Bacterial carriers can be readily produced on a large scale via culturing, eliminating the need for complex chemical synthesis. Guo et al. used these fluorescent bacteria as dual-function probes to develop a novel multiple fluorescent immunosensor for optical sensing and competitive recognition, enabling the simultaneous quantitative detection of three pathogens (*Clostridium difficile*, *L. monocytogenes*, and *E. coli O157:H7*) with a sensitivity below 8.6 × 10^3^ CFU/mL [44].

Fluorescence resonance energy transfer (FRET) involves non-radiative energy transfer between molecules, with extensive applications in biomedicine, chemistry, and materials science [45]. Combining fluorescently labeled donor and recipient molecules with disease markers to detect pathogens by identifying changes in the fluorescent signal has gained prominence in fluorescent biosensing. R M et al. developed a FRET-based fluorescent biosensor for the rapid detection of *Salmonella paratyphi* with a 10 CFU/mL sensitivity [46]. In addition, fluorescence microscopy is vital for pathogen detection due to its high sensitivity, high resolution, and strong specificity. Zhou et al. used fluorescence microscopy to develop an optical biosensor capable of directly identifying multiple pathogens at the cellular level. They created a microenvironment-sensitive aggregation-induced luminescence material to produce a sensitive fluorescent color response to Gram-negative bacteria, Gram-positive bacteria, and fungi. Therefore, fluorescence imaging techniques enable the visual identification and localization of these pathogens at the cellular scale [47].

### 2.3. Optical Biosensor Based on SERS

Surface-enhanced Raman spectroscopy (SERS) is based on Raman scattering and involves molecule absorption on the surfaces of metal or nano-platforms such as gold or silver nanoparticles. Its advantages include simplicity, low consumption, and high sensitivity [48]. SERS is an effective label-free detection tool capable of identifying different pathogenic microorganisms according to the unique Raman characteristics of the molecular compositions (Figure 2C) [49]. However, the limited efficiency of Raman scattering results in suboptimal signal-to-noise ratios and low spectral integrity. Research has shown that employing metal nanostructures as SERS substrates generates hotspots with significant local field amplification on the surface, consequently enhancing the Raman scattering impact of the analyte to achieve satisfactory results. Lyu et al. developed a SERS method for multiple bacterial pathogen detection via the functionalization of AuNPs. The characteristic Raman peaks of the Raman reporter molecules were used for the highly sensitive (as low as 100 copies of target gDNA) multiplex detection of *Mycobacterium smegmatis*, *Staphylococcus epidermidis*, *S. aureus*, and *Pseudomonas aeruginosa* (*P. aeruginosa*). The researchers selected four specific small aromatic molecules as SERS reporter molecules. These molecules have thiol groups and can be directly attached to the surface of gold nanoparticles (AuNPs) through stable Au–S bonds to form a self-assembled monolayer. 4-Mercaptobenzoic acid (MBA) was used for *P. aeruginosa* detection, and its Raman shift wavenumber is 1077 cm^−1^. 7-Mercapto-4-methylcoumarin (MMC) was used for *S. aureus* detection, and its Raman shift wavenumber is 1174 cm^−1^. 5,5′-Dithiobis (2-nitrobenzoic acid) (DTNB) was used for *Staphylococcus epidermidis* detection, and its Raman shift wavenumber is 1343 cm^−1^. 2,3,5,6-tetrafluoro-4-mercaptobenzoic acid (TFMBA) was used for the detection of *M. smegmatis* and its Raman shift wavenumber is 1380 cm^−1^ [50].

Combining two-dimensional membrane nanostructures with precious metal nanoparticles, such as gold and silver, results in the formation of a large number of hotspots, referring to the small gaps between the nanoparticles. These areas significantly enhance the local electromagnetic field, substantially improving the SERS signal intensity. The two-dimensional membrane nanostructure exhibits an extremely high specific surface area capable of efficiently capturing and enriching target pathogens, consequently improving detection accuracy and reliability. Li et al. developed a multiple-SERS-immunochromatography (ICA) biosensor based on the two-dimensional film structure of graphene oxide (GO). This sensor was loaded with Fe_3_O_4_ nanoparticles and two layers of densely arranged AuNPs on the magnetic core, with precisely controlled polyethylenimine (PEI) nanogaps between the nanoparticles. This biosensor successfully captured and detected *S. aureus*, and *Salmonella* at a 10 cells/mL LOD, which was 5- to 10-fold higher than the sensitivity of traditional ICA platforms and exceeded that of AuNP-based platforms by 100- to 455-fold [51]. Wang et al. proposed a three-dimensional film-based SERS tag and introduced it into the lateral flow assay (LFA) system to replace the common spherical SERS nanotag for bacterial detection. Using two-dimensional GO@Au nanosheets as a flexible substrate, the Raman signal was enhanced by the SERS hotspots generated by the built-in nanogaps and Ag satellite particles, enabling the simultaneous detection of *Salmonella*, *E. coli*, *S. aureus*, and *Listeria*, with a 9 cells/mL LOD [52].

Magnetic separation technology utilizes magnetic fields for rapid, effective target molecule separation from complex matrices, minimizing the time and processes required for sample pretreatment. Kearns et al. combined SERS technology and magnetic separation to create an innovative biosensor for multiple bacterial pathogen isolation and identification. Magnetic nanoparticles functionalized with lectin were employed to collect and isolate bacteria from the sample matrix. Then, SERS-active nanoparticles conjugated with strain-specific antibodies were employed for the specific bacterial pathogen identification, successfully detecting methicillin-resistant *S. aureus*, *Salmonella typhimurium* (*S. typhimurium*), and *E. coli* at a minimum concentration of 10 CFU/mL [53].

### 2.4. Optical Biosensor Based on SPR

SPR-based biosensors assess analyte concentrations by evaluating the refractive index changes resulting from target biomolecule or analyte binding to designated recognition sites on metal and dielectric layers [54]. SPR biosensors are extensively utilized in food analysis, environmental monitoring, and medical diagnosis due to their exceptional sensitivity and low-affinity interactions, as well as rapid, label-free response and high accuracy [55,56]. Since AuNPs are highly biocompatible and easily synthesized, they are often used as base materials for SPR optical biosensors for pathogen detection. Wen et al. proposed an AuNP SPR biosensor that utilized the colorimetric changes resulting from the variations in the interparticle distances in AuNPs generated by *E. coli* lysates. Then, a commercial smartphone was used to capture images of the *E. coli*/AuNPs color development and obtain its color signal. The detection range of this approach was 2.44 × 10^5^~1.25 × 10^8^ CFU/mL, with a LOD of 8.81 × 10^4^ CFU/mL [57]. Wei et al. introduced an SPR sensor utilizing AuNPs on the surface of a gold chip to detect fungal toxins. The minimum LODs for deoxysedrine, zearalenone, ochratoxin A, and aflatoxin B1 were 3.26 ng/mL, 7.07 ng/mL, 1.27 ng/mL, and 0.59 ng/mL, respectively, with low cross-reactivity across the four fungal toxins. The experimental data were compared with the high-performance liquid chromatography–tandem mass spectrometry (HPLC-MS/MS) results, exhibiting good consistency [58].

Among these techniques, SERS demonstrates unparalleled sensitivity due to electromagnetic hotspot generation, enabling single-cell detection of Salmonella, while colorimetric methods excel in portability for resource-limited settings. Notably, SPR-based platforms show potential for real-time monitoring of pathogen–host interactions. Table 1 summarized the above-mentioned optical biosensors and their performance.

## 3. Development of Optical Biosensors for Multiple Pathogen Detection

### 3.1. Microfluidics Technology

Microfluidics technology consolidates experimental processes, such as sample preparation, reagent manipulation, biological reactions, and detection, on a miniature chip, offering advantages such as simplicity, cost-efficiency, and rapid detection. Due to their high sensitivity and rapid detection capabilities, optical biosensors display significant potential compared to traditional technologies and are extensively used for multiple pathogen detection.

#### 3.1.1. Paper-Based Microfluidic Optical Biosensors

**Figure 3:**
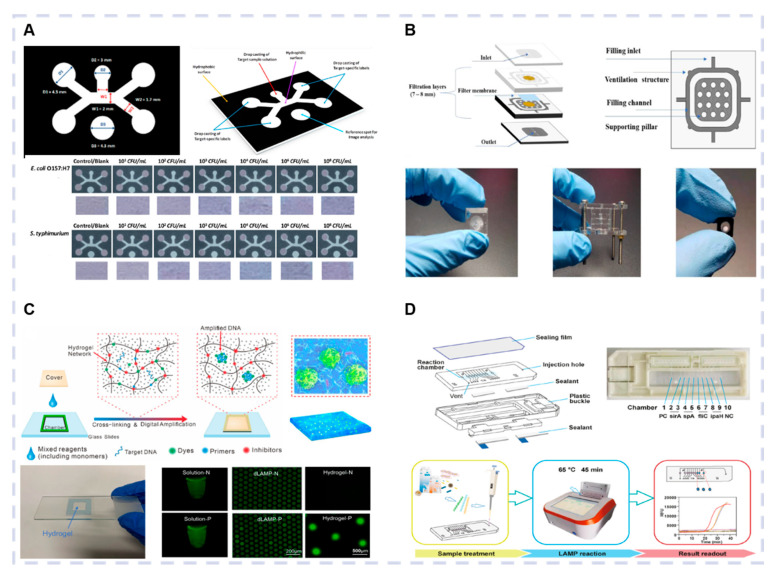
Different microfluidic chip-based optical biosensors. (**A**) Paper microfluidic chip-based optical biosensor. Reproduced with permission from Ref. [59]. Copyright 2022, Elsevier. (**B**) Paper microfluidic chip-based optical biosensor. Reproduced with permission from Ref. [26]. Copyright 2022, Elsevier. (**C**) Paper microfluidic chip-based optical biosensor. Reproduced with permission from Ref. [27]. Copyright 2022, Elsevier. (**D**) Paper microfluidic chip-based optical biosensor. Reproduced with permission from Ref. [28]. Copyright 2022, Elsevier.

Paper-based microfluidic analytical devices have attracted significant attention as a promising analytical platform. Optical biosensors based on paper microfluidic systems are simple, fast, portable, and inexpensive, providing a convenient platform for the on-site identification of multiple pathogens in resource-limited areas. Somvanshi et al. developed an innovative paper-based microfluidic device with a single-input channel capable of simultaneously detecting multiple whole-cell foodborne bacteria, providing quantifiable results via image analysis (Figure 3A). This biosensor simultaneously detected *S. typhimurium* and *E. coli O157:H7* in a linear correlation in a concentration range of 10^2^ CFU/mL to 10^8^ CFU/mL with LODs of 10^3^ CFU/mL and 10^2^ CFU/mL, respectively. LFAs are frequently used for pathogen detection due to advantages such as rapid response, cost-efficiency, high sensitivity, and adequate selectivity [59]. Bai et al. combined recombinase polymerase amplification (RPA) and lateral flow test strip (RPA-LFD) technology to develop a sensitive, rapid method for the detection of *E. coli O157:H7*, *Shigella*, *and Salmonella* in meat. This method accurately distinguished between target pathogens and non-target strains, with DNA LODs of 0.168 fg/μL, 0.72 fg/μL, and 1.25 fg/μL for *E. coli O157:H7*, *Salmonella*, and *Shigella*, respectively, and respective corresponding bacterial concentration LODs of 1.04 CFU/mL, 27.49 CFU/mL, and 48.84 CFU/mL [60]. Song et al. combined a paper-based lateral flow biosensor with a multiplex reverse transcription-recombinant polymerase amplification strategy for the rapid, accurate, and simple detection of multiple viruses. Primers were specifically constructed for the 5’UTR region of the bovine viral diarrhea virus-1 (BVDV-1), the G gene of the bovine epizootic fever virus (BEFV), and the N gene of the bovine respiratory syncytial virus (BRSV). Different fluorescent labels (Dig, ROX, and FAM) were attached to the primers. The three viruses were detected in a range of 10~10^7^ copies/μL, with LODs of 26.2 copies/μL, 24.2 copies/μL, and 25.6 copies/μL for BVDV-1, BEFV, and BRSV, respectively [25].

Paper-based microfluidic optical biosensors are in high demand due to their ability to easily detect specific pathogens via color changes, which are distinguishable by the naked eye, eliminating the need for expensive, complex instrumentation. The primary disadvantage of this approach is low sensitivity, making it challenging to translate a detectable signal into a color readout.

#### 3.1.2. Silicon-Based Microfluidic Optical Biosensors

Polydimethylsiloxane (PDMS) is a high-molecular weight organosilicon compound commonly used for pathogen identification due to characteristics such as electrical insulation, biocompatibility, elasticity, heat resistance, high chemical inertness, and cost-efficiency. Yin et al. developed a PDMS-based microfluidic chip by combining DNA extraction, multiplexed digital recombinase amplification, and fluorescence detection (Figure 3B). It simultaneously detected *Salmonella enterica*, *L. monocytogenes*, and *E. coli O157:H7* within 45 min, providing digital quantitative results with an LOD of 10 bacterial cells for each pathogen [61]. Liu et al. fabricated a custom multi-channel PDMS microfluidic chip for the rapid, simultaneous identification of three bacterial strains. When a sample containing multiple bacterial species was introduced into the chip, the bacteriophages selectively captured the corresponding bacterial strains, which were stimulated at 365 nm using a UV lamp while moving in the flow direction. The luminescent channel lengths were recorded. This approach enabled the quantitative detection of two *S. typhimurium* strains (S. T 14028 and S. T 25928), as well as *E. coli O157:H7* (*E. coli* 44484) [62]. To achieve rapid “sample-in, result-out” detection, Shang et al. developed an optical biosensor using a PDMS microfluidic chip for real-time RPA analysis and nucleic acid extraction. The sensor consisted of a portable signal detector, a detection chip, and a nucleic acid extraction chip. The nucleic acid extraction chip, fabricated using soft lithography, consisted of three layers, with a middle filtration layer containing a glass microfiber membrane to adsorb DNA. The detection chip was preloaded with lyophilized RPA reagents in the reaction chamber during preparation. A 3D-printed signal detector collected fluorescence signals and provided heat and light sources for the chip. This biosensor demonstrated an LOD of 10^3^ CFU/mL for *Salmonella typhi*, *S. aureus*, *P. aeruginosa*, *L. monocytogenes*, *E. coli O157:H7*, and *Vibrio parahaemolyticus*, while the LOD for *Bacillus cereus* and *Cronobacter sakazakii* was 10^4^ CFU/mL [63].

#### 3.1.3. Hydrogel-Based Microfluidic Optical Biosensors

Hydrogels are microfluidic materials with good biocompatibility due to high water content (usually over 90%) and properties similar to biological molecules. Integration with various biomolecules or living bacteria allows the development of biosensors capable of simultaneously detecting multiple targets. Hydrogel-based optical biosensors undergo physical changes, such as swelling or shrinking, when binding to biomolecules, amplifying the subsequent signal and improving the detection sensitivity.

The good permeability and diffusion of chitosan-based hydrogels ensure that the sensor responds rapidly to target substances, which improves detection sensitivity. It integrates multiple detection principles and signal output methods, such as color changes, fluorescence signals, and current changes, to achieve multiple detection and high-sensitivity sensing. Jia et al. developed a colorimetric hydrogel sensor using color coding for multiplex bacterial detection. This method uses chitosan and its derivative N-succinyl chitosan as the hydrogel matrix. Three different colorimetric enzyme substrates (MUD, X-Gal, and PNPG) were grafted onto the hydrogel via EDC/NHS chemical coupling to recognize and respond to the corresponding bacterial enzymes (β-glucuronidase, α-glucosidase, and β-galactosidase). The four test bacteria were successfully distinguished by detecting the enzymatic activity in the bacterial culture medium. For example, *S. aureus* only produced α-glucosidase, while *E. coli DH5α* yielded both α-glucosidase and β-glucuronidase, but not β-galactosidase. The hydrogel sensor achieved the specific, sensitive, rapid multiplex detection of three bacterial enzymes and four bacteria [64]. Cross-linked hydrogels featuring high-density nanoporous systems are widely used for adsorption, release, and separation. Lin et al. developed a biosensor based on cross-linked hydrogel technology. This sensor combined a nanoporous hydrogel with controlled-release functionality and LAMP for the highly accurate quantitative detection of *Salmonella typhi* and *E. coli* in fresh fruits and vegetables within 20 min (Figure 3C). The biosensor demonstrated exceptional sensitivity, capable of single-cell detection [27]. Conventional hydrogels are typically cross-linked into continuous bulk structures, with external dimensions at the millimeter scale or larger and pore sizes at the nanometer scale to allow molecular diffusion. However, bulk hydrogels are unsuitable for certain applications, such as those requiring injectable materials or smaller dimensions. To address these limitations, techniques have been developed to produce hydrogel microparticles (HMPs) (approximately 1~1000 μm). Roh et al. developed a multiplex nucleic acid detection method to encapsulate Cas reactions within biocompatible, encoded HMPs. Each HMP served as an independent Cas reaction vessel. Spatial encoding was used for nucleic acid target differentiation, enabling multiplex detection in a single tube. The system was used to detect high-risk HPV16 and HPV18, with GAPDH serving as a cellular positive control. By selecting specific gRNAs and performing upstream NAAT, the method achieved an LOD of 2 aM [65].

#### 3.1.4. Other Microfluidic Optical Biosensors

Polycarbonate, a thermoplastic engineering material with excellent physical, chemical, and optical properties, is commonly used in microfluidic chips. Polycarbonate displays excellent light transmission, which allows the optical detector to capture the reaction inside the chip. Cao et al. used a polycarbonate material to construct a microfluidic chip for the fluorescent and colorimetric detection of multiple pathogens (Figure 3D). The LAMP-integrated chip enabled the simultaneous detection of *Shigella*, *E. coli O157:H7*, *S. aureus*, and *Salmonella* within 45 min, achieving an LOD of 8 × 10^3^ CFU/mL [28]. Ou et al. used a polycarbonate microfluidic chip for rapid multi-pathogen detection. The chip was injection molded with medical-grade polycarbonate material and contained multiple independent reaction chambers. Specific primers were designed for the conserved genes of several pathogens. The primers were screened, and the reaction conditions were optimized to facilitate the rapid detection of *S. aureus*, *E. coli*, *Klebsiella pneumoniae*, *Shigella*, methicillin-resistant *S. aureus*, and *Candida albicans* [66]. Polymethyl methacrylate (PMMA) is commonly used for multiplex pathogen detection. Li et al. combined a centrifugal PMMA microfluidic chip with real-time fluorescence RPA to simultaneously detect five major pathogenic microorganisms threatening shrimp aquaculture. The chip consisted of a PMMA disk that was 81 mm in diameter and 2.5 mm thick, containing four structural layers divided into four independent sections, each with two units, including sample wells, ball valves, vents, and reaction chambers with a volume of 5 μL. The chip was preloaded with primers and probes, along with internal positive and negative controls. This microfluidic platform facilitated the analysis of six genetic targets within a minimal reaction volume of 5 μL, enabling the detection of five pathogenic microorganisms in shrimp [67].

### 3.2. Nucleic Acid Amplification Technology

Amplifying target nucleic acid sequences is crucial for detecting trace pathogen nucleic acids. NAAT incorporates numerous pairs of specific primers into the same reaction system to simultaneously detect and amplify multiple targets. Furthermore, integration with microfluidic chip technology can facilitate the automated “one sample in, multiple results out” detection process.

#### 3.2.1. Multiplex PCR-Based Optical Biosensors

**Figure 4:**
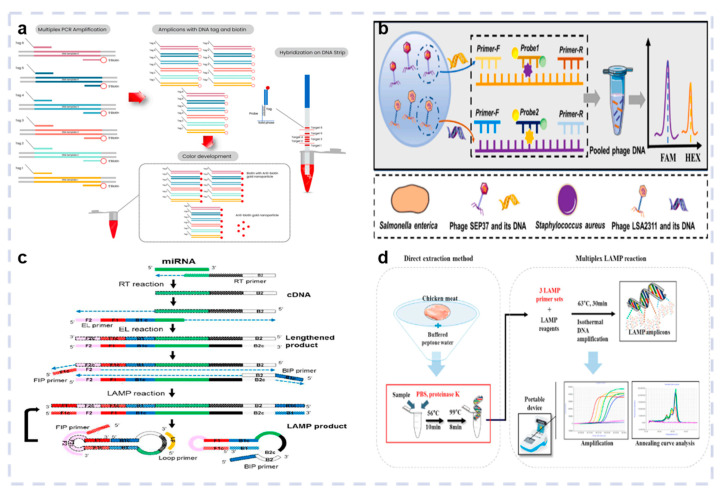
Multiplex PCR-based optical biosensor. (**a**) Principle of the PCR reactions. Reproduced with permission from Ref. [29]. Copyright 2022, Elsevier. (**b**) Schematic diagram of multiplex PCR and multiplexed detection of foodborne pathogens. Reproduced with permission from Ref [70]. Copyright 2023, Elsevier. Multiplex LAMP-based optical biosensor. (**c**) Principle of the PCR reactions. Reproduced with permission from Ref. [71]. Copyright 2019, American Chemical Society. (**d**) Schematic representation of the direct triplex LAMP assay developed for food sample testing. Reproduced with permission from Ref. [72]. Copyright 2021, Elsevier.

The introduction of PCR has enabled highly sensitive, precise nucleic acid sequence amplification, leading to significant biotechnology and biomedical engineering advancements. Contrary to traditional PCR, multiplex PCR (mPCR) incorporates two or more primer pairs into a single PCR reaction system to amplify numerous nucleic acid fragments (Figure 4a). Furthermore, mPCR can detect multiple target pathogens simultaneously in one reaction system, which is faster and less expensive. Li et al. proposed a propidium monoazide-based mPCR to specifically and accurately detect live *Salmonella enteritidis*, *E. coli O157:H7*, and *L. monocytogenes* in fresh vegetables, with an LOD of 10 CFU/g [68]. Yuan et al. established an mPCR method to identify five virulence-related genes in the mulberry wilt pathogen. Specific primers were designed using the pleD, yjfP, pelY, ampD, and ripW genes. This method can specifically detect *Klebsiella oxytoca* complex, *Klebsiella pneumoniae* species complex, *Enterobacter cloacae* complex, *Pantoea ananatis*, and *Ralstonia pseudosolanacearum*, with a minimum detection concentration of 1.87 × 10^3^ CFU/mL (DNA = 2.45 pg/μL) [69]. However, mPCR exhibits poor sensitivity for bacterial detection due to the complex reaction matrix. Therefore, recent studies have combined phage amplification with mPCR to improve sensitivity. Huang et al. used this strategy to identify *S. aureus* phage LSA2311 and *Salmonella* phage SEP37 (Figure 4b). Specific TaqMan probes and primers were constructed, while the DNA of the phage progeny was identified via mPCR to facilitate the quantitative detection of *Salmonella* and *S. aureus*. The LOD of this approach reached 10 CFU/mL, with a detection time below 4 h [70].

#### 3.2.2. Multiplex LAMP-Based Optical Biosensors

To address the challenges of thermal cycling in PCR, various isothermal amplification methods have been applied for multiplex pathogen detection, including LAMP, RPA, and rolling circle amplification (RCA). These isothermal amplification techniques enable nucleic acid amplification at a constant temperature, showing considerable promise for developing portable, cost-efficient, and highly sensitive optical biosensing devices.

LAMP is extensively employed for isothermal nucleic acid amplification due to its high specificity and efficiency. It requires only a single Bst DNA polymerase (derived from *Bacillus stearothermophilus*, known for its excellent strand displacement activity) and a set of four target-specific primers, consisting of two internal and two external primers (Figure 4c). Multiplex LAMP-based optical biosensing techniques were developed for the simultaneous detection of multiple pathogenic targets. Kim et al. proposed a novel triple-multiplex LAMP detection method for the identification of *Salmonella* genus, subspecies I, and *S. typhimurium* (Figure 4d) using three sets of LAMP primers that target specific genes in these bacteria. The annealing curves of the individual LAMP reactions were analyzed, allowing the differentiation of multiple targets within 60 min [72].

#### 3.2.3. Digital NAAT-Based Microfluidic Optical Biosensors

**Figure 5:**
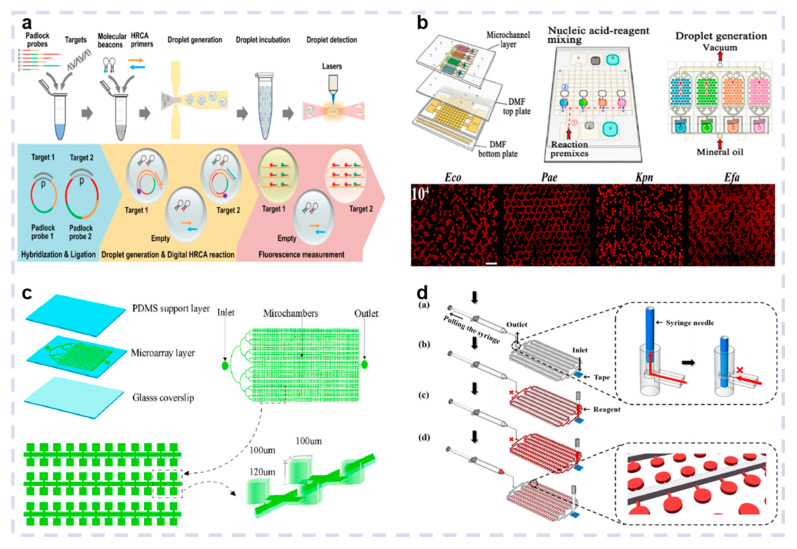
Droplet digital NAAT-based optical biosensor. (**a**) Principle of the PCR reactions. Reproduced with permission from Ref. [31]. Copyright 2020, Elsevier. (**b**) Schematic diagram of integrated ddNAAT microfluidic device for uropathogen rapid detection. Reproduced with permission from Ref. [79]. Copyright 2024, American Chemical Society. Chip digital NAAT-based optical biosensor. (**c**) Layout of the chip for cdNAAT. Reproduced with permission from Ref. [81]. Copyright 2021, Elsevier. (**d**) Schematic representation of cdNAAT for bacterial detection. Reproduced with permission from Ref. [80]. Copyright 2024, Elsevier.

Lab-on-a-chip (LOC), also known as micro-total analysis system, is a miniaturized analytical platform that integrates microfluidics, microelectronics, biosensors, and other technologies [73]. It has the advantages of small size, automatic operation, rapid response and low reagent consumption and has shown potential in many applications, including medical diagnosis, genomics and proteomics research, analytical chemistry, environmental monitoring and biohazard detection [74]. The LOC system greatly shortens the analysis time by integrating sample pretreatment, reaction, separation, and detection steps and realizes the rapid detection of biological objects (such as bacteria, viruses, cells, DNA, etc.). Optical biosensors, as its key detection modules, can be integrated into the LOC system to achieve high-sensitivity optical detection of biological objects [75]. For example, optical biosensors based on Micro-Ring Resonator can be integrated with microfluidic channels to detect changes in biomolecule concentrations [76]. Some optical biosensors (such as surface plasmon resonance sensors) can realize real-time, label-free detection of biomolecular interactions, avoiding the influence of the labeling process on biological activity. In addition, by integrating different optical components and detection methods, multi-parameter simultaneous detection of biological samples can be achieved [77]. Advances in isothermal amplification technology combined with lab-on-a-chip systems have resulted in methods such as digital NAAT, which enables absolute nucleic acid. Advancements in microfluidics and lab-on-a-chip technologies have yielded methods such as digital NAAT, which enables absolute nucleic acid molecule quantification in pathogens, single intact cells, and even viruses without requiring calibration curves. Typically, microliter samples are divided into separate droplets using microfluidics chips, each capable of encapsulating a single target for digital amplification. Digital NAAT is divided into droplet-based digital NAAT (ddNAAT) and chip-based digital NAAT (cdNAAT) according to their micro-reaction chambers. The ddNAAT reaction chambers are generated via droplet microfluidics technology and consist of microliter or nanoliter droplets dispersed in an oil phase. Choi et al. developed a multiplex droplet digital RPA method using a microfluidic chip to segregate and rapidly combine the master mixture and initiator in a closed droplet. The high heat and droplet mass transfer facilitated accurate amplification within 30 min, showing high linearity (R^2^ > 0.999) in a target RNA concentration ranging between 5 copies/μL and 2500 copies/μL. This method was highly specific (R^2^ > 96%) in detecting three human coronaviruses [78]. Zhang et al. developed a multiplex digital NAAT platform using droplet-digital-ratio fluorescence encoding (Figure 5a). This platform employed unique two-fluorophore combinations to encode each nucleic acid target using a padlock probe-based nucleic acid detection assay. The system successfully detected the nucleic acids of six major pathogens associated with sexually transmitted infections, including *Chlamydia trachomatis*, *Treponema pallidum*, Human Immunodeficiency Virus, *Neisseria gonorrhoeae*, Herpes Simplex Virus, and *Mycoplasma genitalium*. This approach provides a straightforward solution for highly multiplexed digital NAAT [31]. Digital microfluidics (DMF) typically employs the electrowetting-on-dielectric (EWOD) technique for precise droplet control, facilitating the dispensing, driving, merging, and splitting of droplets in the nanoliter to microliter range. Xie et al. integrated digital and droplet microfluidics to develop a rapid detection device for multiplexed nucleic acid analysis (Figure 5b). This system comprised a bottom DMF module and a top droplet microfluidics module, which was combined with LAMP technology for the simultaneous detection of *E. coli*, *P. aeruginosa*, *Klebsiella pneumoniae*, and *Enterococcus faecalis*. The entire process was completed within 75 min in a detection range of 9.43 × 10 to 2.86 × 10^4^ copies/μL [79]. CdNAAT employs microfluidics technology to form tiny reaction chambers on the chip for the absolute quantification of nucleic acid molecules (Figure 5c). Quan et al. used negative pressure actuation to design a DMF chip with 800 microwells, each with a volume of 20 nL, capable of encapsulating up to one bacterium (Figure 5d). The entire chip was fabricated via photolithography and PDMS molding, with 16 50-well arrays and a vacuum system to ensure liquid filling and bacterial encapsulation. This method yielded an average accuracy of 97.72% and an LOD 63 CFU/mL for the identification of *Salmonella*, *Bacillus cereus*, *S. aureus*, and *E. coli O157:H7*. Compared to the traditional standard plate count method, the DMF platform significantly increases accuracy and reduces detection time from 24 h to 7 h [80].

Combining microfluidic chips with various NAAT technologies has attracted significant research attention due to advantages such as integration, automation, and miniaturization. The dispensing capabilities of microfluidics are critical for digital NAAT implementation, regardless of whether it is a chip- or droplet-based system.

### 3.3. Nanomaterial Technology

The rapid rise of nanotechnology has increased the utilization of metallic, carbon, and magnetic nanomaterials in optical biosensors to improve the sensing capabilities of detection and identification systems. Nanomaterials enhance the ability of optical biosensors to bind to specific biological receptors, providing an alternative for the sensitive and specific identification of various pathogens.

#### 3.3.1. Metal Nanomaterial-Based Optical Biosensors

**Figure 6:**
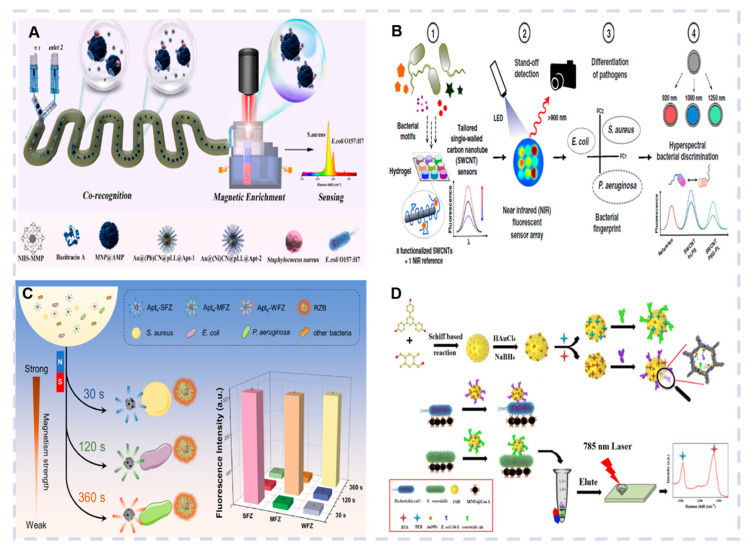
(**A**) Metal nanomaterial-based optical biosensor for multiplexed detection of pathogens. Reproduced with permission from Ref. [85]. Copyright 2024, Elsevier. (**B**) Carbon nanomaterial-based optical biosensor for multiplexed detection of pathogens. Reproduced with permission from Ref. [86] Copyright 2020, Nature. (**C**) Magnetic nanomaterial-based optical biosensor of multiplexed detection of pathogens. Reproduced with permission from Ref. [87] Copyright 2021, Elsevier. (**D**) Optical biosensor based on other nanomaterials of multiplexed detection of pathogens. Reproduced with permission from Ref. [88]. Copyright 2022, Elsevier.

Metal nanoparticles have been extensively studied for biomedical applications due to their narrow particle size distribution, high biocompatibility, and stability. The characteristic strong light scattering properties of metal nanoparticles can be used as signal amplifiers to improve the detection sensitivity of optical biosensors. AuNPs are commonly used for pathogen detection due to their high stability and minimal degradation. Some studies have combined AuNPs and colorimetric strategies for rapid, accurate multiplex pathogen detection. Park et al. developed an optical biosensor by combining AuNP-based colorimetric detection with LAMP. AuNPs prepared via D-glucose oxidation were used to detect the *Enterococcus faecium* and *S. aureus* foodborne pathogens, with LODs of 10 CFU/mL and 100 fg/μL, respectively [82]. Dayalan et al. proposed a gold nanostar-based SERS biosensor for specific and sensitive quantitative food pathogen identification. In this study, 4-mercaptobenzoic acid was used as a Raman reporter molecule and linker to attach vancomycin to the surfaces of GNSs. The proposed SERS biosensor achieved LODs of 8.2 CFU/mL and 5.7 and CFU/mL for *E. coli* and *S. aureus*, respectively [83]. Zopf developed an optical biosensor that used AuNP arrays as a sensing platform to detect the DNA of various pathogens via SPR. The sensor successfully identified the DNA sequences of multiple fungal pathogens and achieved reversible binding via chemical methods (10 mM HCl) [84]. Huo et al. introduced a SERS biosensor employing AuNPs as SERS tags for multiple pathogen detection (Figure 6A). The AuNPs were modified with aptamers, while encapsulating Prussian blue analogs (PBAs) served as silent SERS tags. This biosensor combined a flexible microfluidic system and a magnetically controlled slider device for *S. aureus* and *E. coli O157:H7* detection in a concentration range of 50~1600 CFU/mL, exhibiting a linear relationship between the Raman intensity and concentration and yielding LODs of 14 CFU/mL and 18 CFU/mL, respectively. When applied to milk and chicken breast samples, the sensor yielded recovery rates between 85.6% and 112.3% and relative standard deviation (RSDs) values ranging from 1.5% to 8.6%. Compared with ELISA, the sensor demonstrated a RSD between 7.5% and 4.3% [85].

#### 3.3.2. Carbon Nanomaterial-Based Optical Biosensors

Carbon nanomaterials, such as carbon dots (CDs), semiconductor quantum dots (QDs), and carbon nanotubes (CNTs), are commonly used for pathogenic detection due to their extraordinary optical, electrical, and physicochemical properties. CDs are a nano-fluorescent material that has attracted considerable attention in recent years. Compared with conventional fluorescent materials, CDs display low toxicity and excellent optical characteristics, such as a high PL quantum yield and photostability, as well as tunable multicolor properties. Wang et al. presented a new approach for identifying pathogenic microorganisms using single concentration-dependent CDs. Microwave heating was used to synthesize the CDs, which eliminated the need for complicated purification processes. The PL emission wavelengths were adjusted by simply altering the concentration of the solution. Concentration-dependent CDs and different fluorescence spectra were used for the multicolor biological imaging, separation, and identification of *E. coli O157:H7*, *S. aureus*, and *L. monocytogenes* [89].

QDs are essential probes used for biosensor construction due to their exceptional optical properties. As fluorescent labels, QDs increase brightness and offer superior photostability compared to traditional fluorescent dyes, enabling sensors to generate distinct fluorescence signals even in the presence of low-concentration targets. Cheng et al. developed a fluorescent optical biosensor using carbodiimide coupling chemistry to prepare CdSe/ZnS QD-conjugated antibodies. This biosensor simultaneously detected multiple Gram-positive bacteria by analyzing the fluorescence signals of the QDs. In optimized conditions, the LODs for *S. aureus*, *Bacillus cereus*, and *L. monocytogenes* were 18 CFU/well, 3 CFU/well, and 36 CFU/well, respectively [90]. CNTs demonstrate significant potential for pathogen detection due to their unique structures and exceptional properties. Nissler et al. developed a single-walled CNT (SWCNTs)-based near-infrared (NIR) fluorescent nanosensor, featuring a hydrogel array comprising nine distinct sensors, each designed to respond to different bacterial characteristics (Figure 6B). The system employed remote NIR imaging technology at distances ≥25 cm to successfully identify multiple clinically relevant bacteria, including *P. aeruginosa* and *S. aureus* [83].

In summary, optical biosensor arrays utilizing carbon nanomaterials exhibit considerable potential for multiple pathogen detection due to their outstanding optical signaling capabilities. However, several challenges remain that require resolution. The precise photoluminescence mechanisms of carbon nanomaterials, as well as the modulation of their fluorescence properties to develop ideal sensing units, require further investigation. Synthetic methods should be optimized to produce stable, high-performance carbon nanomaterials for practical applications.

#### 3.3.3. Magnetic Nanomaterial-Based Optical Biosensors

In recent years, magnetic nanomaterials have attracted considerable attention due to their significant potential for multiple pathogen detection. These materials are primarily composed of magnetic metals, such as cobalt, nickel, and iron, as well as their related oxides (e.g., γ-Fe_2_O_3_ and Fe_3_O_4_), exhibiting exceptional properties, including superparamagnetism, high monodispersity, and rapid separation capability. Multiplex detection enables the simultaneous identification and quantification of multiple analytes within a single test. However, current fluorescence-based methods face challenges such as spectral crosstalk interference, a limited multicolor label range, and complicated design and synthesis. Li et al. developed a strategy for simultaneously detecting multiple pathogens using fluorescence quantification and magnetic resolution-based separation. Mercaptopropyltrimethoxysilane, magnetic γ-Fe_2_O_3_ nanoparticles, and fluorescent QDs were used for self-assembly via metal coordination interactions to obtain multifunctional nanoprobes exhibiting distinct magnetic responses and excellent fluorescence properties. The method efficiently separated and detected the *E. coli O157:H7* and *S. typhimurium* pathogens within 2 min in a broad linear range, displaying low LODs [91].

Magnetic encoding fluorescent nanoparticles offer novel opportunities for the simultaneous detection of multiple analytes. Wu et al. developed a magnetic-encoded nanoparticle fluorescence biosensor using multiple nucleic acid aptamers (Figure 6C). Fe_3_O_4_ nanoparticles were embedded in a zeolitic imidazolate framework-90 (ZIF-90) to fabricate Fe_3_O_4_@ZIF-90 composites with varying magnetic intensities. By functionalizing the surface of the Fe_3_O_4_@ZIF-90 composites with specific aptamers, the biosensor was effective in simultaneously detecting multiple coexisting bacteria, including *P. aeruginosa*, *E. coli*, and *S. aureus* in milk samples [92].

#### 3.3.4. Optical Biosensors Based on Other Nanomaterials

Metal–organic frameworks (MOFs) have garnered considerable attention in recent years for biosensing applications due to their exceptional properties. MOFs possess remarkable physicochemical properties, including large specific surface areas, a high adsorption affinity, and conjugated π-electron systems, which enable outstanding fluorescence quenching efficiency. Chai et al. examined the impact of various synthesis parameters on the morphology and size of MOFs, successfully optimizing the conditions for the production of uniform MOF particles with a high quantum yield. These MOFs selectively bonded to Gram-positive bacterial cell walls. This caused aggregation and enabled the rapid differentiation between Gram-positive and Gram-negative bacteria within 20 min [93]. Covalent organic frameworks (COFs) represent porous crystalline materials consisting of organic building blocks connected via strong covalent bonds. COFs exhibit highly ordered structures, tunable porosity, and exceptional chemical and thermal stability, making them promising materials for various applications, including biosensing, drug delivery, catalysis, and gas storage. Yang et al. used COFs to develop a novel SERS immunosensor for the multiplex detection of foodborne pathogens (Figure 6D). The sensor was synthesized via a Schiff-base reaction to produce TBDP, followed by in situ reduction to prepare AuNP-doped TBDP nanoparticles. Raman reporter molecules and antibodies were subsequently loaded onto TBDP@Au to create Raman tags. This immunosensor successfully detected *E. coli* and *Salmonella enteritidis*, with an LOD of 10 CFU/mL [85].

Lanthanide chelates are complexes consisting of chelating ligands and rare-earth lanthanide ions, exhibiting unique fluorescence properties that make them superior to other fluorescent labels. They feature extended fluorescence deterioration times, narrow emission spectra, and substantial Stokes shifts. Jin et al. combined europium nanoparticles with RPA technology to develop a lateral flow strip biosensor for the simultaneous detection of *E. coli O157:H7*, *Salmonella* spp., *Streptococcus suis*, *S. aureus*, and *L. monocytogenes.* This method achieved a detection sensitivity of 10¹ CFU/mL and demonstrated strong linear correlations for all five pathogens, with R² values exceeding 0.96 [94].

Despite the development of several nanoparticles, there is a general lack of standardized methods for their preparation, particularly for large-scale synthesis at an industrial level. Additionally, the toxicity, biocompatibility, and environmental sustainability of nanomaterials pose significant challenges.

## 4. Application of Optical Biosensors for Multiple Pathogen Detection in POCT

**Figure 7:**
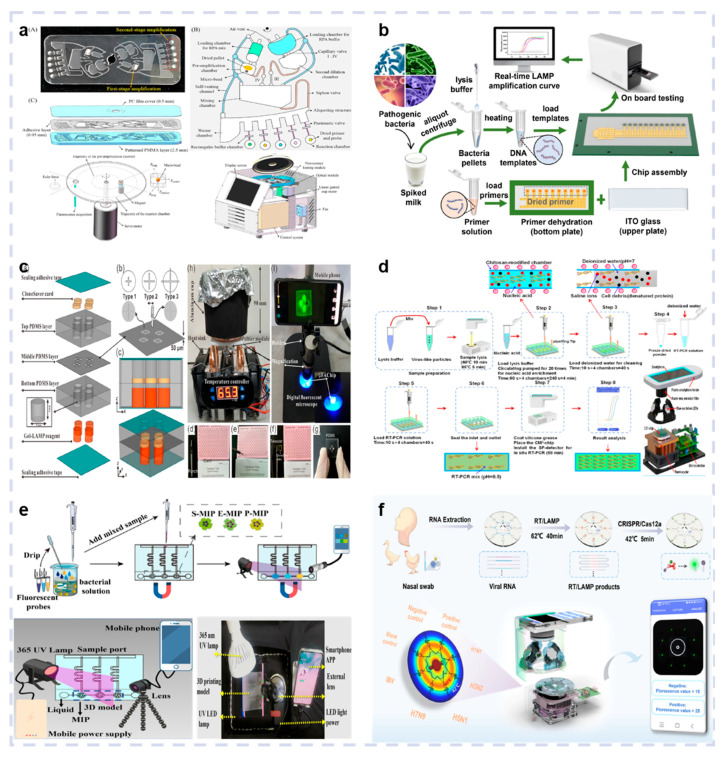
Non-smartphone integrated optical biosensors. (**a**) Principle of the PCR reactions. Reproduced with permission from Ref. [95]. Copyright 2024, Elsevier. (**b**) Schematic diagram of the portable optical biosensor device combines DMF with real-time LAMP technology. Reproduced with permission from Ref. [96]. Copyright 2022, Elsevier. Smartphone integrated optical biosensors. (**c**) Illustration of the POCT platform for multiple bacterial detection based on a finger-driven microfluidic chip. Reproduced with permission from Ref [97]. Copyright 2021, Elsevier. (**d**) Schematic representation of the direct triplex LAMP assay developed for food sample testing. Reproduced with permission from Ref. [98]. Copyright 2021, Elsevier. (**e**) Principle of the PCR reactions. Reproduced with permission from Ref. [99]. Copyright 2019, Elsevier. (**f**) Schematic representation of the centrifugal microfluidic disk spatially encoded integrated smartphone biosensor device. Reproduced with permission from Ref. [100]. Copyright 2024, Elsevier.

The previously described technological advancements have resulted in the miniaturization, portability, and integration of optical biosensors into handheld or smartphone platforms, enhancing their utility for multiple pathogen detection in POCT. The integration of microfluidic chips, automated sample processing systems, and intelligent data analysis algorithms enables the implementation of biosensors to identify various diseases during POCT. Dong et al. developed a portable analyzer using a two-stage centrifugal microfluidic chip (Figure 7a), which integrated a servo motor, a precise temperature control module, and a real-time fluorescence module for acquisition and analysis to control chip rotation, heating, and fluorescence signal detection. The results show that combining two-stage amplification with active mixing mode yielded the highest detection sensitivity, successfully identifying different gene fragments of African swine fever virus (ASFV), with a minimum LOD of 3.5~7.5 copies/test [95]. Xie et al. combined DMF with real-time LAMP technology to develop a portable optical biosensor device (Figure 7b). Spiked milk samples were used for verification, facilitating the successful simultaneous detection of four pathogens, with the lowest detection concentrations of 2.7 × 10^3^ CFU/mL, 3.5 × 10^3^ CFU/mL, 2.6 × 10^3^ CFU/mL, and 4.6 × 10^3^ CFU/mL for *E. coli*, *S. typhimurium*, *S. aureus*, and *L. monocytogenes*, respectively. The device employed dehydrated primers to facilitate multiple pathogen detection, demonstrating significant potential for food safety testing [96].

Optical biosensors frequently employ smartphones as a vital component due to their integrated cameras and microprocessor capabilities. Chen et al. developed a POCT platform for multiple bacterial detection using a finger-driven microfluidic chip (Figure 7c). The chip consisted of three PDMS layers bonded by two layers of tape, including a top layer for sample preparation, a bottom layer for prolonged LAMP reagent storage, and a middle layer containing finger-driven valves. In POCT applications, on-chip LAMP reactions and imaging are performed on a miniature Peltier heater and a portable smartphone-based fluorescence imaging system, respectively. In optimized conditions, this system simultaneously identified multiple bacteria, including *Salmonella*, *P. hauseri*, and *E. coli O157:H7*, with high selectivity and sensitivity (as low as 1.6 copies) [97]. Yang et al. developed a POCT device by combining a minimalist chitosan-modified microfluidic chip (CM3 chip) and a smartphone (Figure 7d). The CM3 chip consisted of a PDMS layer, a glass layer, and a sealing plug. A mobile phone app was used to control the temperature, obtain the fluorescence images, and analyze the data. The practicality of the system was verified via artificially prepared saliva samples infected with H5N6 and IAV, yielding LODs of 5 × 10^2^ copies/mL and 3.24 × 10^2^ copies/mL, respectively [98]. Yin et al. employed smartphone image processing, fluorescent probes, and magnetic separation to create a fluorescent sensor for sensitive multiple pathogen detection. A microchannel device was constructed using 3D printing and highly transparent resin (Figure 7e). This device was integrated with an external lens and a smartphone to obtain a fluorescent sensor for autonomous multiple pathogen detection. Tetraphenylethylene derivatives and highly specific aptamers were employed to synthesize three fluorescent probes, which elicited different fluorescent colors from *P. aeruginosa*, *E. coli*, and *S. aureus*. The enrichment properties of molecular imprinting materials can be used for simultaneous bacterial separation and detection. Finally, the red, green, and blue (RGB) analysis function of the smartphone was used for real-time on-site detection with a 10^2^ CFU/mL sensitivity and a 40 min detection duration [99]. Yin et al. developed a centrifugal microfluidic disk, spatially encoded, integrated smartphone biosensor device, which simultaneously identified five influenza virus subtypes, including influenza A virus H1N1, H3N2, H5N1, and H7N9, as well as influenza B virus within 45 min, with an LOD of 10 copies/μL (Figure 7f). Its application for influenza virus subtype detection was verified by evaluating 16 negative and 22 positive clinical samples [100].

Future optical biosensors are expected to be smaller, lighter, and potentially portable or wearable. These sensors will enable rapid on-site testing, especially for disease detection in areas with limited medical resources or in emergency situations.

## 5. Conclusions and Prospects

In recent decades, the prevalence of environmental pathogenic microorganisms has become a significant concern, posing a risk to human health, the economy, and public safety. This paper examines the current status of optical biosensors for the identification of multiple pathogens, identifying challenges and future development trends from three perspectives: microfluidic technology, NAAT, and nanomaterials. The utilization of microfluidics technology facilitates the efficient processing and analysis of trace samples, thereby reducing detection times and enhancing the efficacy of detection. When combined with optical sensors, the system is able to automate sample pretreatment, nucleic acid amplification, and detection, thereby accelerating the identification of multiple pathogens. The employment of bespoke primers or probes enables the precise identification of diverse pathogens through the utilization of nucleic acid amplification technology. When combined with optical sensors, it can efficiently detect multiple pathogens and significantly reduce detection times. The combination of nanomaterials with biorecognition molecules, such as antibodies and aptamers, has been demonstrated to facilitate targeted recognition and enrichment of specific pathogens. This approach has been shown to enhance the specificity and sensitivity of detection. The application of optical biosensors in the detection of multiple pathogens, in combination with microfluidic devices, nucleic acid amplification technology, and nanomaterials, demonstrates unique advantages but also faces certain limitations. For instance, the channel dimensions of microfluidic chips are ordinarily diminutive and thus susceptible to obstruction by impurities or cell detritus in the sample, thereby compromising the accuracy and reliability of the test results. Nonspecific amplification or contamination has been observed to occur during nucleic acid amplification, resulting in false positive results and compromising the reliability of detection. The cost of producing high-quality and high-purity nanomaterials is high, which limits their wide application in the detection of multiple pathogens.

The anticipated progress in biosensor technology for the simultaneous detection of multiple pathogens presents a promising research area. Optimizing the sensor array design and signal processing algorithm will enable the simultaneous detection of a broader variety of pathogens, markedly enhancing the efficiency and accuracy of the identification process. Moreover, the integration of advanced biometric recognition technology with molecular biology methods is expected to enhance pathogen typing and quantitative analytical ability of optical biosensors, consequently establishing a solid foundation for precise disease diagnosis and treatment. Incorporating machine learning algorithms into optical biosensors may improve the accuracy of complex test results, particularly in the context of developing artificial intelligence technology [101]. The capacity of machine learning to accurately predict complex biological information has rendered it a valuable tool in the development of optical sensors for multiple pathogen detection [102]. Moreover, the incorporation of diverse technologies will enhance the sophistication of optical biosensors. Extensively integrating optical biosensors with electronic technology, information technology, and the Internet of Things will enable functionalities such as remote monitoring, real-time data analysis, and intelligent early warning. This will significantly expand the use of sensors and augment their practical value in addressing public health incidents.

In conclusion, optical biosensor technology represents a multidisciplinary collaboration between fields such as computational science, mechanical engineering, materials science, analytical chemistry, and optics. Collaborative efforts among specialists across several disciplines aim to develop more sophisticated, practical, and cost-effective optical biosensors for the detection of diverse pathogens to enhance food safety, environmental monitoring, and public health.

## Figures and Tables

**Table 1:** Summary of optical biosensors and their performance.

Technique	Principle	Sensitivity	Multiplex Capacity	Portability	References
Colorimetric	Enzymatic/chemical-induced color shift	10–10^6^ CFU/mL	3–5 targets	High (naked-eye readout)	[39]
Fluorescence	QDs/CDs as fluorescent probes	10–10^3^ CFU/mL	5–8 targets	Moderate (UV excitation)	[43,44]
SERS	Hotspot-enhanced Raman signals	1–10 CFU/mL	>10 targets	Low (laser required)	[53]
SPR	Refractive index changes	10^2^–10^4^ CFU/mL	3–4 targets	Moderate (chip-based)	[57]

## Data Availability

The data analyzed during the study are available from the corresponding author upon reasonable request.
